# Deploying a robotic positive psychology coach to improve college students’ psychological well-being

**DOI:** 10.1007/s11257-022-09337-8

**Published:** 2022-07-11

**Authors:** Sooyeon Jeong, Laura Aymerich-Franch, Kika Arias, Sharifa Alghowinem, Agata Lapedriza, Rosalind Picard, Hae Won Park, Cynthia Breazeal

**Affiliations:** 1 MIT Media Lab, Cambridge, MA, USA; 2 Computer and Information Sciences College at Prince Sultan University, Riyadh, Saudi Arabia; 3 Department of Communication at Pompeu Fabra University, Barcelona, Spain; 4 Estudis d’Informàtica, Multimèdia i Telecomunicacióat Universitat Oberta de Catalunya, Barcelona, Spain

**Keywords:** Socially assistive robot, Positive psychology, Well-being

## Abstract

Despite the increase in awareness and support for mental health, college students’ mental health is reported to decline every year in many countries. Several interactive technologies for mental health have been proposed and are aiming to make therapeutic service more accessible, but most of them only provide one-way passive contents for their users, such as psycho-education, health monitoring, and clinical assessment. We present a robotic coach that not only delivers interactive positive psychology interventions but also provides other useful skills to build rapport with college students. Results from our on-campus housing deployment feasibility study showed that the robotic intervention showed significant association with increases in students’ psychological well-being, mood, and motivation to change. We further found that students’ personality traits were associated with the intervention outcomes as well as their working alliance with the robot and their satisfaction with the interventions. Also, students’ working alliance with the robot was shown to be associated with their pre-to-post change in motivation for better well-being. Analyses on students’ behavioral cues showed that several verbal and nonverbal behaviors were associated with the change in self-reported intervention outcomes. The qualitative analyses on the post-study interview suggest that the robotic coach’s companionship made a positive impression on students, but also revealed areas for improvement in the design of the robotic coach. Results from our feasibility study give insight into how learning users’ traits and recognizing behavioral cues can help an AI agent provide personalized intervention experiences for better mental health outcomes

## 1 Introduction

College students’ mental health issues are on the rise (Benton et al. 2003). In 2018, about 85% of college students in the USA reported feeling “overwhelmed by all [they] had to do” and nearly 43% reported “feeling so depressed that it was difficult to function” at least once within the last 12 months (Association et al. 2018). Depression and anxiety are reported as the top hurdles to academic success (Breslau et al. 2008) and have a long-lasting effect on students’ occupational (Ettner et al. 1997) and social (Kessler et al. 1998) outcomes as well. Higher education institutions are making efforts and expanding their mental health services in order to improve students’ mental well-being. Yet, several systemic and personal barriers still exist for these students, such as cost, lack of knowledge about resources, social stigma, etc., and hinder them from seeking and receiving help (Ebert et al. 2019; Marsh and Wilcoxon 2015).

Recent developments in interactive technologies for mental health aim to close this gap by increasing accessibility to the resources and services that were traditionally only available through human clinicians. Some help with tracking and monitoring patients’ conditions easily through mobile or wearable devices (Vidal et al. 2012; Chum et al. 2017). Others offer interactive psycho-education or intervention content through electric or mobile devices that can be accessed any time at the users’ request (Fitzpatrick et al. 2017). However, most of their interactions with users are limited to the health-related tasks. For instance, Woebot,^1^ a mobile chatbot that provides cognitive behavioral therapy, can hold conversations with its users about their mental health and techniques to improve it, but does not deliver a daily news report or play interactive games with them.

Unlike human therapists or clinicians whom patients typically only meet in the clinical/therapeutic care context, personal electronic devices can be physically with us outside of the healthcare context. We believe an interactive agent can build a positive relationship and rapport with its users by interacting with and engaging them in both health-related tasks and in other contexts. Positive working alliance between the agent and its user could even amplify the effect of the intervention the agent offers. It has been shown that rapport between clinicians and patients is a predictor of not only improved health outcomes, but also patients’ adherence to the prescribed regimen and satisfaction with the treatments (Qina’au and Masuda 2020; Fuertes et al. 2007; Wampold 2015). Strong rapport also helps patients to cope with depression and stress (Qina’au and Masuda 2020) and allows the clinician and the patient to collaborate and agree on treatment goals and expectations, and easing the long-term behavioral changes required for successful health outcomes (Qina’au and Masuda 2020).

Based on the social psychology literature, several works in human–robot interaction and human–computer interaction have found that artificial agents can use verbal and nonverbal cues, such as facial expressions, empathetic feedback, back-channeling, prosody, and intonation, to build rapport with people (Lucas et al. 2017; Riek et al. 2010). It was also shown that shared experiences can create a sense of familiarity, trust and mutual understanding between the user and agent (Bickmore and Picard 2005). However, there has not been any work that investigates how the rapport between a robot and its users can enhance the effectiveness of the robot’s mental health interventions.

Personality traits have also been identified as a predictor of adherence to medical regimen (Christensen and Smith 1995) and mental health (Hooker et al. 1998; Packard et al. 2012; Villieux et al. 2016; Lamers et al. 2012). High conscientiousness has been positively correlated with longevity (Hill et al. 2011), ability to cope with daily stress (Bartley and Roesch 2011), and response to treatment (Quilty et al. 2008). Low neuroticism and high extraversion has been found to be correlated with high psychological well-being (Kokko et al. 2013). We acknowledge that early personality research has typically focused on a small portion of population (e.g., men in the military Gibby and Zickar 2008), and the measures for personality traits in our study might not be reflective of minority individuals’ life experiences (Arshad and Chung 2021). However, the Mini-IPIP used our study has been adapted for several other cultures for reliability and validity [e.g., Spanish (Martínez-Molina and Arias 2018), French (Martínez-Molina and Arias 2018), Malay (Leong et al. 2019), and Portuguese (Oliveira 2019)].

Based on these prior works, we designed a robotic coach that provides seven positive psychology interventions and a variety of useful skills (e.g., weather forecast, interactive games and music streaming). It can also engage in pro-social behaviors (e.g., proactive greetings, pleasantries and calling users by their names) to enhance the rapport with the users. This allows the robot to not only deliver mental health interventions, but also serve as a helpful companion. Our novel intervention framework that combines the robot’s positive psychology based interactions with its pro-social companionship was evaluated through an on-campus deployment study with 35 college students. We designed a feasibility study to investigate the following research questions:

**RQ1** Can a robotic positive psychology coach improve college students’ psychological well-being?

**RQ2** How do students’ personality traits affect the efficacy of the intervention?

**RQ3** What effect does students’ rapport with the robot have on the intervention outcomes?

**RQ4** Can we predict the intervention outcomes by observing students’ behaviors during their interaction with the robot?

In our previous work (Jeong et al. 2020), we found associations between our robotic intervention and positive changes in students’ well-being, mood and readiness to change behavior for better well-being, as well as the association between students’ personality traits and the well-being outcomes after students completed the intervention with the robot. In this paper, we further explore the effect of students’ rapport with the robot on the intervention outcomes and analyze the relationship between the students’ behavioral cues (e.g., facial expressions, body posture, etc.) during the interaction and the intervention outcomes. Our analyses showed that people’s therapeutic alliance with the robot was correlated with improved motivations for better psychological well-being, and students’ vocal/facial/gestural expressions were correlated with their changes in intervention measures. Our work gives insights into how interactive technologies could leverage information about users’ traits and behaviors to better tailor and personalize health interventions for better adherence and health outcomes, and what behavioral cues to detect and respond to in order to assess the progress of the robot-mediated intervention on a moment-to-moment basis instead of relying on people’s self-report data.

## 2 Related works

Section 2.1 summarizes existing interactive technologies developed for improving people’s well-being in order to highlight the novel contribution of our robotic coach in offering positive psychology interactions along with a variety of other features and its companionship. Next, Sect. 2.2 describes patient-related factors found to impact the effectiveness of the intervention, and in particular how a patient’s personality traits influence the treatment process. Section 2.3 provides a brief overview of positive psychology and why we chose to use interventions based on positive psychology in our study. Finally, Sect. 2.4 reviews how behavioral cues could inform people’s mental states and could further be used to predict the therapeutic outcomes.

### 2.1 Interactive technologies for well-being

Personal devices and technology have created new avenues for people to access services and resources for mental health. Several mobile applications allow people to request help, monitor progress, and receive interventions. Mobile applications, such as Headspace^2^ and Calm,^3^ offer meditation and breathing exercises designed to reduce stress and depressive moods. Chatbots, e.g., Woebot (Fitzpatrick et al. 2017), offer interactive experiences for mental health interventions and therapies. Wearable devices, e.g., Fitbit, use built-in sensors to collect information and detect user behaviors in order to provide timely feedback.

Embodied conversational agents (ECA) and social robots can also engage people in natural interactions by leveraging their embodiment and social behaviors (Sebastian and Richards 2017; Gardiner et al. 2017; Scoglio et al. 2019; Riek 2016; Lane et al. 2016; Suganuma et al. 2018). For instance, a low-fidelity social robot developed by Björling et al. (2020) was shown to reduce teens’ stress level through self-disclosure activities. In comparison with human care providers, these agents can lessen the feeling of stigma and heighten the perception of anonymity to promote more honest and candid responses from patients. However, most of these agents have a single “skill” and are not equipped to help their users in other tasks. On the other hand, our robotic coach is designed to not only offer mental health interventions but other useful skills to engage with users in various contexts.

### 2.2 Factors influencing intervention outcomes

According to the aptitude–treatment interaction (ATI) research, no treatment or intervention works equally well for all patients, and therapists should identify a treatment plan that matches each individual patient’s specific characteristics for optimal therapeutic outcomes (Snow 1991). Tailored and personalized interventions have been shown to improve patients adherence and health outcomes (Garbers et al. 2012; Han et al. 2009; Mosnaim et al. 2015), and predictors of patient outcomes could inform clinicians to develop and prescribe the most suitable treatment for each individual patient (Barber 2007). Predictors of outcome variables that have been commonly analyzed are patient-related predictors and therapist-related predictors. Patient-based predictor variables commonly examined include demographics, diagnoses, personality traits, patient expectancies or symptom severity, among others (Barber 2007; Delsignore and Schnyder 2007). For instance, a literature review that explored 35 studies focusing on the relationship between patient expectancies and psychotherapy found a modest but direct relationship between outcome expectancies and therapeutic improvement (Delsignore and Schnyder 2007). Therapist characteristics that have been explored as possible predictors of working alliance include among others, therapists’ experience, training, and skill (Hersoug et al. 2001).

Moreover, a few studies investigated the relationship between personality traits and adhering to lifestyle changes (e.g., diet or exercise), treatments (e.g., medication) or psychotherapy (e.g., Cognitive Behavioral Therapy). For example, as reviewed in Driesenaar et al. (2018), some studies found that adhering to treatments has a negative correlation with neuroticism and a positive correlation with conscientiousness, while no relation with the other three traits. Studying healthy lifestyles in young adults, Steptoe et al. (1994) found a positive correlation with extraversion, and a negative association with neuroticism. Interestingly, personality traits were a predictive factor of risk of dropout for Internet-based cognitive behavioral therapy, where low dropout risk was associated with extraversion, and high risk with openness (Schmidt et al. 2019).

### 2.3 Positive psychology

Positive psychology focuses on enabling people to flourish through enhancing their personal strengths and the positive aspects of their lives (Seligman and Csikszentmihalyi 2014). In comparison with clinical psychology, which primarily focuses on treating negative mental and emotional pathology, positive psychology is an ideal intervention to enhance the well-being of non-clinical populations. Additionally, both clinical and non-clinical populations have been shown to benefit from positive psychology based interventions. Individuals diagnosed with clinical depression and individuals who do not suffer from any psychological disorders both experience reduced symptoms of depression and increased psychological well-being after engaging with psychotherapy interventions based on positive psychology (Seligman et al. 2006).

### 2.4 Interpreting behavioral cues for mental health interventions

Verbal and nonverbal cues have been widely used to infer or detect people’s mental states. For instance, acoustic signals (e.g., pitch, energy, spectral features and formants) can be used to infer people’s emotions in conversations, therapy, call centers, etc. (Swain et al. 2018). Temporal speech behaviors, such as speech rate and pauses, have been investigated in the detection of hesitation, confidence, anxiety (Corley and Stewart 2008) and even neurological disease (Alvar et al. 2019). Understanding affect is crucial for developing alliance/empathy and achieving successful therapeutic interactions (Avdi and Evans 2020).

Facial expressions are another way for humans to express and interpret affective and cognitive states. Analysis of facial expression showed strong potential in assessing the level of rapport in dyad (Wang and Gratch 2009) and group interactions (Müller et al. 2018). A recent study done by Shidara et al. (2020) found mood improvement was correlated with facial expressions observed during cognitive therapies with a virtual agent.

Nonverbal behaviors are also expressed through body movement as surveyed in Kleinsmith and Bianchi-Berthouze (2013), Karg et al. (2013), Zacharatos et al. (2014), Stephens-Fripp et al. (2017) and Noroozi et al. (2018). Expressive movement is categorized into four types: communicative (e.g., gestures), functional (e.g., walking), artistic (e.g., choreography), and abstract (e.g., arm lifting), where a single or a combination of these types represents an affect (Karg et al. 2013). For example, anxiety is linked to expanded limbs and torso, fear is linked to elbows bent, and shame is linked to bowed trunk and head (Kleinsmith and Bianchi-Berthouze 2013). Ekman and Friesen (1969) also showed that self-soothing body movement (such as touching one’s neck, face or body and holding/crossing one’s arms) could indicate anxiety. Head movement and orientation indicate not only emotions, but also indicate affirmation and affiliation in an interaction as surveyed in Noroozi et al. (2018).

In this study, we designed a robotic coach protocol for positive psychology intervention, where we account for several factors of participant-related (e.g., personality trait, current well-being states) and therapist-related (e.g., building rapport and agent persona). We analyze these factors quantitatively and qualitatively with regards to the intervention outcomes. Participants’ verbal and nonverbal behaviors observed during the interactions with the robot were also analyzed to investigate the relationship between observed behavioral cues and the efficacy of the robot intervention. Results from such analyses will offer valuable insights for the interactive agents to adapt and tailor their behavior and interventions based on users’ personality, preferences, and behaviors during the interactions.

## 3 Robotic positive psychology coach

### 3.1 Robot stations for long-term deployment study

**Fig. 1:**
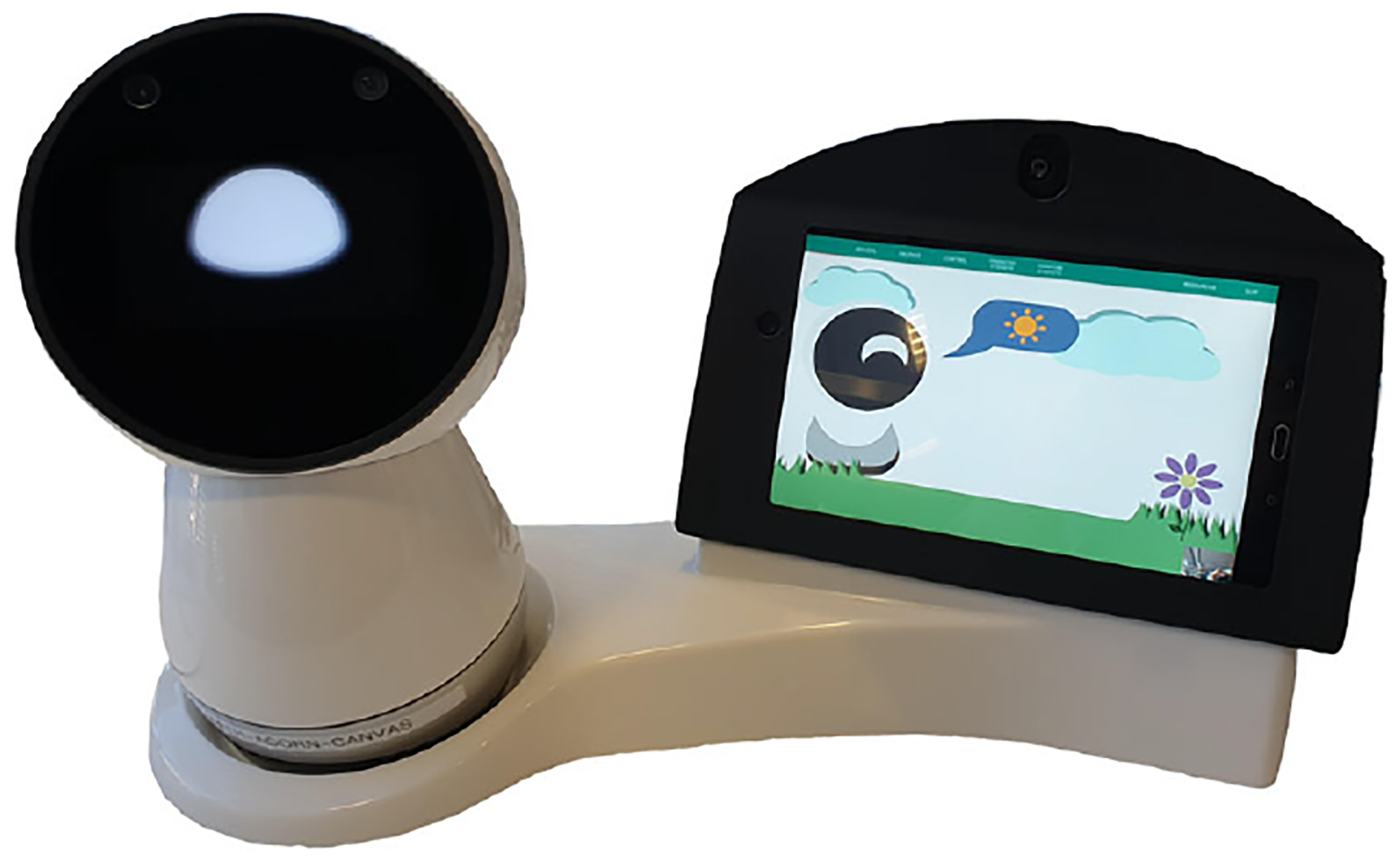
We designed a robot station that integrates a Jibo robot, Android tablet, and Raspberry Pi with a USB camera for our on-campus deployment study

For our on-campus deployment study, we designed a robot station (Fig. 1) that holds a Jibo robot,^4^ Samsung Galaxy tablet, Raspberry Pi, and USB camera (Jeong et al. 2020). It is 20 × 9 × 14 in. and weighs about 16 lb. The robot can orient itself to look at its user and gaze toward the Android tablet when referring to content displayed on the tablet screen. The Android tablet is used to display visual content or receive users’ input through a touch screen interface during the interaction. The Raspberry Pi inside the station is connected to a high-resolution and wide-angled camera and can record video and audio to capture the interaction data. We designed the base of the station in a curved shape to minimize the surface area and resemble the overall shape of the robot. The station only needs one power cord to be connected, which enables a quick and easy installation for at-home deployment studies.

### 3.2 The Jibo robot

Jibo is a social robot that can interact with its users through expressive movements, verbal communications and touch gestures. It has a three-axis motor system that allows it to face any direction and use a variety of expressive movements. Jibo’s two on-board cameras and microphone array allow it to locate and orient itself to users when interacting with them. It is equipped with several basic skills, such as weather forecast, information retrieval, interactive games, physical exercises, and jokes, in addition to the custom positive psychology skill developed for our study. Unlike other voice user interfaces (e.g., Siri or Alexa) that are passive, Jibo can proactively greet and prompt users to engage in interactions. For instance, if Jibo sees its user in the morning, it might say “Hi [*Name*], did you have a good night sleep?”. Or, it sometimes asks whether the user wants to play a *Word of the Day* game. Jibo also has an animate idle behavior that involves blinking, looking around its surroundings and self-play behavior.

### 3.3 Software architecture

**Fig. 2:**
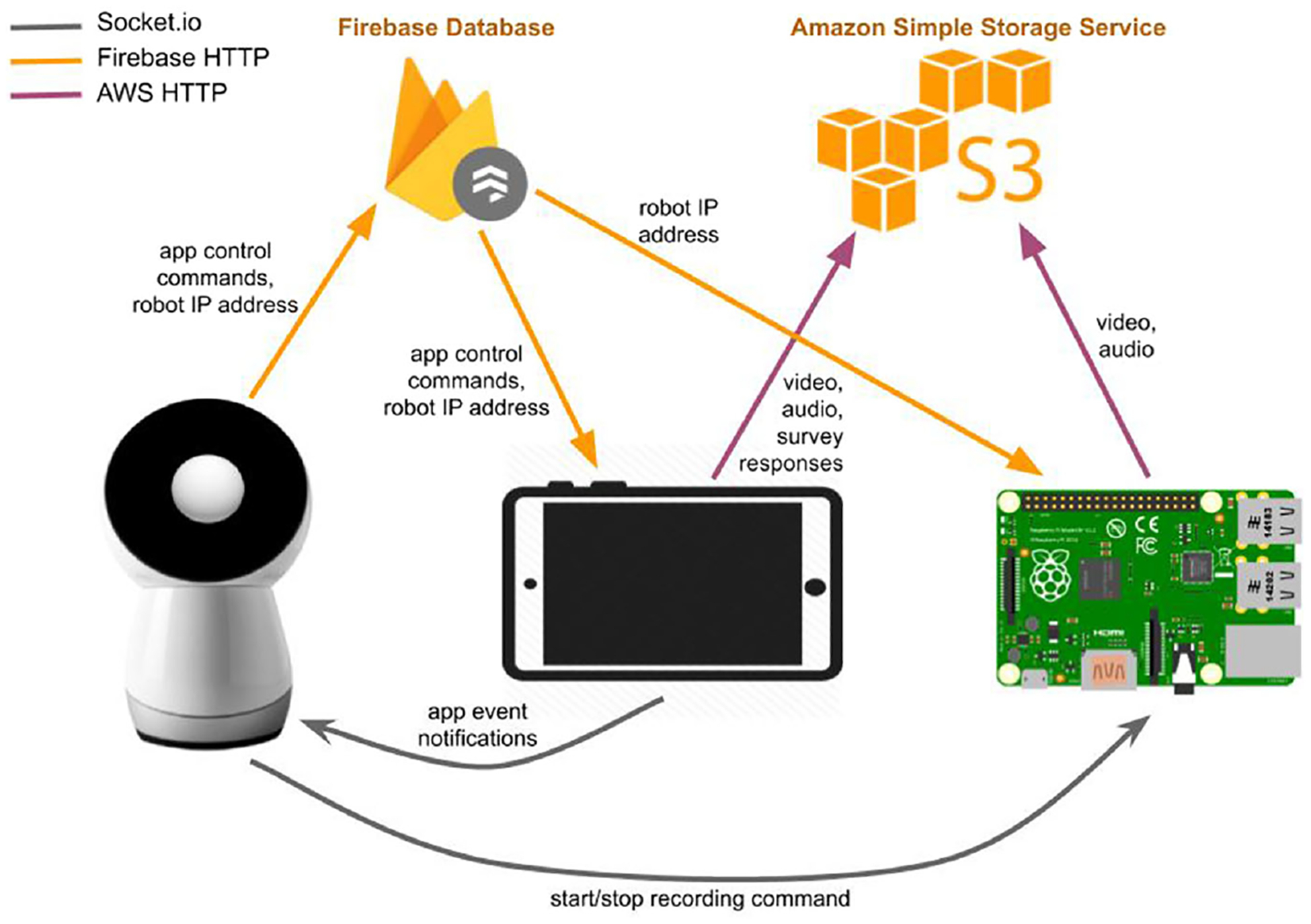
Software architecture for the robot station. The Jibo robot, Android tablet, and Raspberry Pi communicate with one another via Socket.IO and Firebase Messaging. The recorded interaction data are uploaded to the S3 after each session is completed

We developed a custom positive psychology skill for Jibo that provides seven distinct interactions. This positive psychology skill acts as a master controller for the Android tablet and the Raspberry Pi on the robot station. The Jibo robot, the Android tablet and the Raspberry Pi communicate with one another via Firebase Cloud Messaging (FCM)^5^ and Socket.IO.^6^ The system diagram is presented in Fig. 2.

Each Android tablet subscribes to a unique FCM topic, which is set as the robot’s serial name, e.g., Clay-Data-Caraway-Velvet. When the positive psychology skill on Jibo is launched, the skill publishes an FCM message with its serial name as the topic, and automatically starts the Android application designed for the study. The Android application creates a directory on the local device to temporarily store the video/audio recordings and any other study related data. During the interaction session, the robot publishes FCM messages to control screen views of the Android application. The robot also sends its own private IP address with the FCM message, and the Android application uses the received IP address to create a Socket.IO connection with the robot to communicate any user input made on the tablet back to the robot. At the end of the positive psychology session, the robot publishes an FCM message to close the Android app.

A Firebase Realtime Database^7^ is used to match a Raspberry Pi MAC address with its corresponding robot’s name and IP address. The robot updates a pair of its name and IP address every hour while it was turned on to ensure the database contains the latest IP address information. With the retrieved robot IP address, the Raspberry Pi waits for the connection from the robot positive psychology skill. Once a connection is made, the Raspberry Pi communicates with the robot via Socket.IO.

At the end of each positive psychology session, the Android tablet and the Raspberry Pi both close their positive psychology applications and upload the interaction data to an Amazon Simple Storage Service (S3)^8^ bucket. After the uploads are completed, the data on the local devices are deleted.

In order to protect participants’ data privacy, each participant was assigned a unique participant ID, and all study data (e.g., interaction video data, survey responses and interview recordings) were labeled by this participant ID or the robot’s serial name. Data stored on Firebase Realtime Database or Amazon S3 and did not contain personal information about the participants, such as their name, age, gender, or contact information.

### 3.4 Designing positive psychology sessions with Jibo

For this study, we designed seven interventions based on a variety of well-known principles and exercises from the field of positive psychology (Bryant and Veroff 2017; Peterson and Seligman 2004; Seligman et al. 2005, 2006; Rashid 2015; Aymerich-Franch and Johnston 2019). In **session 1**, Jibo introduces what positive psychology is and the general structure of the sessions. In **session 2**, Jibo explains the concept of character strengths, which are positive, trait-like capacities for thinking, feeling and behaving in ways that benefit oneself and others (Niemiec 2013). Participants are then asked to identify five strengths that best reflect who they are and are told these chosen strengths are their *signature strengths* (Peterson and Seligman 2004). Jibo invites participants to think of a time when they used one of their signature strengths. In **session 3**, Jibo encourages participants to create a plan to “use [their] signature strengths in a new way”(Seligman et al. 2005; Mongrain et al. 2012). They are also guided through a visualization activity to help solidify their intent to implement this plan. Jibo encourages participants to execute the plan before the next session as homework. In **session 4**, Jibo checks whether the participants completed the assignment from the previous session, and introduces the concept of gratitude and its importance. Participants are guided to complete the “three good things in life”exercise (Seligman et al. 2005), in which they write down and reflect on three things that went well that day and why they went well. In **session 5**, the participants learn about the “gratitude visit”exercise (Seligman et al. 2005) and are asked to practice it as homework. For this exercise, they are asked to write a short letter or email to someone that they FEEL like they have never properly thanked. In **session 6**, Jibo asks participants to reflect about their experience with the “gratitude visit,”and introduces the savoring exercise. Participants are asked to plan a savoring activity and to carry it out before the next session as homework (Bryant and Veroff 2017). In **session 7**, participants reflect about the savoring activity and the positive emotions they felt during the activity. Jibo reviews and summarizes all of the interventions introduced in the study and asks the participants to rate how helpful each activity was for them. Finally, Jibo thanks the participants for completing all sessions with it and concludes the interaction. The seven positive psychology sessions are summarized in Table 1.

Several design considerations were made when developing interaction script. First, Jibo uses informal and casual language during the positive psychology sessions even though it is presented as a coach. Jibo has a playful and light personality and was designed to appeal to a broad range of population regardless of age or gender. Since the commercial features of Jibo were available for study participants to use, we designed the positive psychology session to also use informal language, expressive animations, and lighthearted anecdotes. This allowed Jibo’s persona in the positive psychology skill to be consistent with its persona in the rest of the skills.

In addition, each positive psychology session was designed to be a collaborative process. Most of the sessions had a significant amount of intervention concepts and materials to be explained and instructed. In order to keep users engaged even during long expository segment of the session, the robot often asked users for confirmation and minor feedback during the interaction.

Jibo uses rule-based parsers to classify users’ intention, such as yes/no, accept/refusal, or positive/negative responses. For instance, Jibo would engage in a small talk at the beginning of each positive psychology session by asking something like “Hey [name], how’s your day going?”. If the participant’s answer is classified as positive, it might respond “I’m really glad to hear that.” On the other hand, if the response is negative, it would say something like “Oh, sorry to hear that. I’m sure things will get better soon.” The robot responded differently based on the participant’s utterance but the interaction followed a prescripted dialogue flow for the most part. We designed Jibo’s responses to human utterance carefully considering its limited natural language understanding capability, and often used generic responses, such as “thanks for sharing” or “that’s great to hear” for more open-ended responses.

On average, each session has twelve prompts for free response: two for long open-ended response for participants’ thoughts, opinions, or ideas, and ten for simple yes/no responses or confirmations for the next step. These frequent prompts made the overall interaction as an interactive and collaborative experience, rather than a one-sided interaction.

Lastly, each session was designed to take about five minutes in order to accommodate college students’ busy schedules. Many students have several responsibilities and are often over-scheduled with academics, extracurricular activities, and social lives. We hoped that the students would perceive the daily robot session as a pleasant break from their busy daily routines, rather than another burdensome task on their to-do list. With these design considerations, we developed seven unique interactions that introduce positive psychology exercises to improve college students’ well-being.

### 3.5 Data analyses

In order to find answers for our first research question (**RQ1**), we evaluated the changes in college students’ psychological well-being, mood, and motivation for better well-being after interacting with our robotic coach. In addition, the impacts of students’ personality traits and therapeutic alliance were tested in order to answer the second and the third research questions, respectively (**RQ2** and **RQ3**). In addition, we conducted exploratory behavioral analyses to investigate the relationship between verbal/nonverbal cues and the intervention outcome to address our fourth research question (**RQ4**). We anticipated from these analyses that we will be able to develop adaptive mental health applications in the future.

## 4 Feasibility study

An on-campus deployment study was conducted in order to evaluate the efficacy of our robotic positive psychology coach. This study was conducted in accordance with the recommendations of the MIT Committee on the Use of Humans as Experimental Subjects.

### 4.1 Participants

Forty-two undergraduate students signed up to participate in the study through an e-mail advertisement. The total number of participants who completed all study procedures were thirty five (age *M* = 18.94, SD = 1.43; 27 female, 7 male, and 1 other). Nineteen of them were freshmen, eight were sophomores, four were juniors, two were seniors and two were fifth year students. There were twelve students who identified themselves as Asian/Pacific Islander, fourteen as White, two as Hispanic or Latino, one as Black or African American and six as multiracial. All participants signed an informed consent form prior to the study.

### 4.2 Study procedure

**Fig. 3:**
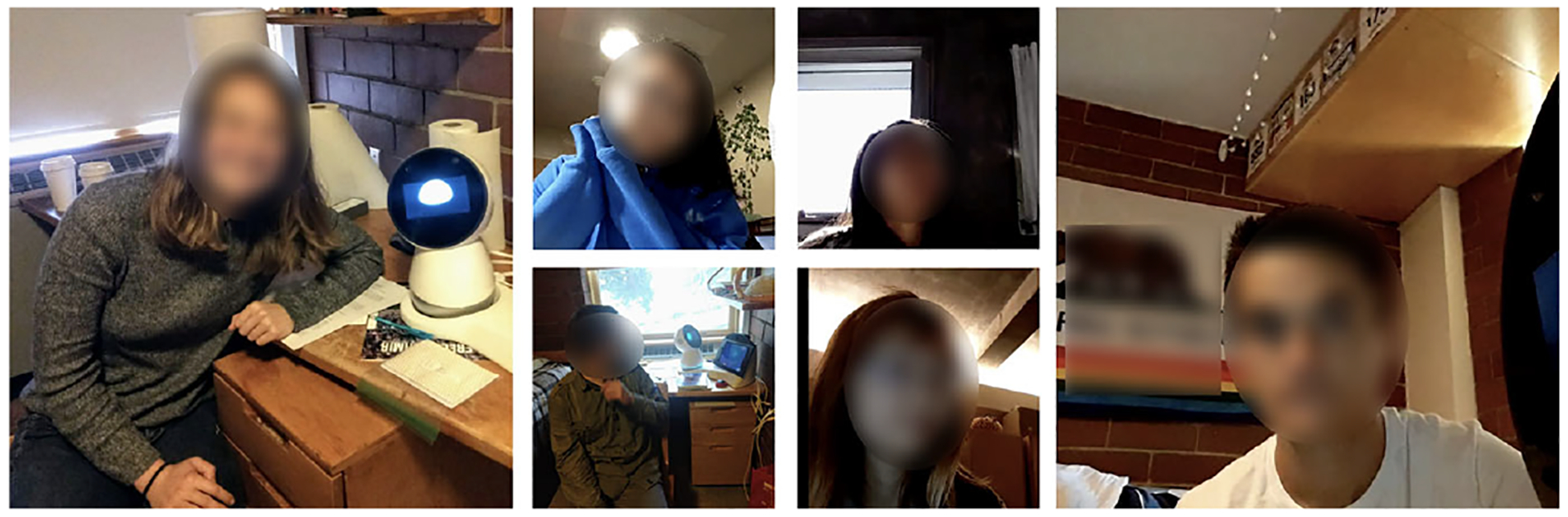
Robotic positive psychology coaches were sent to on-campus dormitory rooms to measure the efficacy of social robot in improving college students’ psychological well-being. All participants in the figure have given consents to non-commercial use their images for academic publishing

An e-mail advertisement was sent out through an undergraduate students’ mailing list in order to recruit study participants. Once a consent to participate in the study was obtained, a robot station was delivered to the participant’s dormitory room (Fig. 3). A set of questionnaires were administered to measure participants’ personality traits, psychological well-being, mood and readiness to change health behavior for better well-being as a pretest. Further details on the self-report questionnaires follow in Sect. 4.3. Then, participants were given a brief orientation on the overall study procedure and proper usage of the robot station. They were informed how to use the robot’s wake word (“Hey, Jibo”), how to start/stop the robot’s positive psychology skill, and how to make the robot go to sleep or turn around for their privacy. We asked the participants to complete the positive psychology session with the robot once a day any time during the day they found suitable. Further descriptions on other robot skills were given, e.g., weather report, interactive games, general Q&A skill, music, etc. Participants were left to use these skills at their leisure while completing the daily positive psychology sessions with the robot.

At the end of the study, participants filled out a set of questionnaires regarding their psychological well-being, mood, readiness to change behavior and working alliance with the robot. We also conducted a semi-structured interview for more open-ended feedback on the overall study procedure, co-living experience with a robot, and suggestions for improving our system and robot intervention.

There were twenty robots available for the study, which is fewer than the total number of study participants. Thus, our deployment study was done in two phases. Twenty students were chosen for the first round of study from the sign-up form, and participated in the study on early October. When participants from the first deployment completed the study and the robot station was returned, additional students not chosen in the first round were contacted to begin the study protocol around late October/early November for the second round of deployment.

### 4.3 Data collection

#### 4.3.1 Self-report measures

Prior to the study, participants were asked to complete the Mini-IPIP (International Personality Item Pool) scale (Donnellan et al. 2006) to measure their personality traits (conscientiousness, agreeableness, neuroticism, openness to experience and extroversion). A set of questionnaires were administered before and after the study in order to measure the pre-to-post change of participant’s psychological well-being, mood and readiness to change. The Ryff’s Psychological Well-being Scale (RPWS) (Kállay and Rus 2014) was used to measure participants’ psychological well-being with six sub-components (autonomy, environmental mastery, personal growth, positive relations with others, purpose in life and self-acceptance) (Political and Research 2010). The Brief Mood Introspection Scale (BMIS) (Mayer and Gaschke 1988) assesses participants’ overall mood by having them select their current state in relation to 16 mood-related adjectives. An adapted version of the Readiness to Change Ruler (Hesse 2006 was administered to assess participants’ willingness and motivation to make behavior changes for better psychological well-being.

At the end of all robot interactions, participants completed the Working Alliance Inventory-Short Revised (WAI-SR) (Munder et al. 2010) and a short survey on their satisfaction with the intervention sessions. The WAI-SR assessed the extent of collaboration between a clinician/therapist and a client on three sub-scales (bond, goals and tasks). We used the WAI-SR to measure the rapport the participants built with the robot. The intervention satisfaction survey was a set of 7-point Likert-scale questions that evaluated how helpful the participant felt each positive psychology intervention was for their psychological well-being.

#### 4.3.2 Behavioral data

The positive psychology sessions with Jibo were video-/audio-recorded via the Android tablet and the USB camera connected to the Raspberry Pi on the robot station. Whenever the system was recording, the Android application displayed a live video feed at the bottom right corner on the tablet screen to notify the recording status. Once the session with the robot was completed, the tablet application uploaded the recorded data with other relevant meta data to S3, and the local data were deleted from the table.

After the study, the audio data were transcribed by a professional transcription vendor. The video data and the audio transcriptions were used to analyze participants’ verbal and nonverbal behavior cues.

#### 4.3.3 Post-study interview

We conducted a semi-structured interview with each participant at the end of the study to gain more qualitative feedback on the robot. During the interview, participants shared their overall experience with the study, things they liked/disliked about Jibo and the intervention sessions. We also asked whether they would continue practicing the positive psychology activities the study, and whether there were any features they would like to see in robots in the future. The whole list of the interview questions is given in Table 3. All interview responses were audio-recorded and transcribed for qualitative analysis.

## 5 Data analysis methods

### 5.1 Statistical analysis

The changes in participants’ psychological well-being (RPWS), mood (BMIS) and readiness to change health behavior (Readiness Ruler) were analyzed by conducting paired sample *t* tests. We analyzed the relationship among participants’ Big Five personality traits by calculating Pearson’s correlation coefficients. Mixed ANOVAs were conducted to investigate the effects of personality traits (Mini-IPIP) on the pre-to-post change of intervention outcomes. For this personality intervention outcome analysis, participants were clustered into high and low groups based on personality trait metrics using the K-means clustering algorithm (*k* = 2). Students’ *t* tests were used to assess the impact of personality traits on participants’ rapport with the robot (WAI-SR) and satisfaction with each positive psychology intervention. A mixed ANOVA was used to evaluate the effect of participants’ personality traits (Mini-IPIP) on their ratings for the positive psychology interventions introduced in the study. We conducted Pearson’s correlation tests to understand the relationship between students’ working alliance with the robot (WAI-SR) and the efficacy of the intervention (change in RPWS, BMIS, Readiness Ruler). Finally, we explored the relationship between participants’ verbal/nonverbal cues and the intervention outcomes (change in RPWS/BMIS/Readiness Ruler and WAI-SR) by calculating Pearson’s or Spearman’s correlation coefficients based on the normality the behavior features.

### 5.2 Behavior annotation and features

#### 5.2.1 Manual annotation of behaviors

Based on the recorded interaction video data, two trained annotators labeled participants’ behavioral, emotional, and attention engagement. The definitions of these engagements are as follows:

- **Behavioral engagement** to be “on-task” or “off-task,” where behavioral cues such as eye gaze direction, head orientation, body gestures, and responsiveness indicates such engagement to the session.
- **Emotional engagement** are any facial and/or body expression that indicates satisfaction, confusion, or boredom.
- **Attention (cognitive) engagement** to indicate focused, idle, or distracted attention to the session content. Attention engagement differs from behavioral engagement at the cognitive level. That is, sometimes the participant would be on-task (not distracted) but not giving a full cognitive focus—i.e., idle.

First, both annotators were asked to annotate 30 sessions from 5 participants to calculate the agreement before continuing with the remainder of the sessions of the other 30 participants. The agreement of the two annotators was measured using Kappa for continuous variables (Mandrekar 2011), which is commonly used when the duration of annotation is variable between the annotators. Kappa agreement value of less than 0 indicates no agreement, 0–0.20 a slight agreement, 0.21–0.40 a fair agreement, 0.41–0.60 a moderate agreement, 0.61–0.80 a substantial agreement, and 0.81–1 a almost perfect agreement. The agreement of the two annotators from the 30 sessions was 0.80, which is considered substantial, and therefore, further sessions were annotated by only one of the annotators.

#### 5.2.2 Verbal and nonverbal features

We automatically extracted verbal and nonverbal features from speech transcripts and audio/video data, and studied the correlations between the features and the self-report measures.

For each participant’s turn, we extracted high-level functional (statistical) **acoustic features** using OpenSmile (Eyben et al. 2010). A recent comparison of acoustic features for Affect investigations (Eyben et al. 2015) showed a high performance using the minimalistic feature set carefully selected features, which is now widely used for verbal behavior analysis. For this paper, we used the extended Geneva Minimalistic Acoustic Parameter Set (eGeMAPS), which contains 88 parameters, that includes frequency parameters (pitch, jitter, and formants), energy parameters (shimmer, loudness, and harmonic-to-noise ratio (HNR)), and spectral parameters (including mel-frequency cepstral coefficients (MFCC). The extracted parameter set include voiced and unvoiced segments. Voiced segments are calculated from the phonemes that require vibration of the vocal cords, such as vowels, while unvoiced segments are phonemes that do not entail the use of the vocal cords, such as stop consonants /p/. In OpenSmile extraction process, there was no restriction of the minimum duration of voiced and unvoiced segments, which means that unvoiced segments include both pauses and consonants phonemes. These acoustic features capture several prosodic behaviors from the speech, such as intensity (identify stress) and monotone (identify boredom, tiredness, and depression), to name a few examples.

From the videos recorded from the tablet, we extracted **facial expressions and facial activities** in terms of Action Units using the Affdex SDK (McDuff et al. 2016). We extracted 30 facial emotions and facial activities every 5 frames. These features were then summarized using statistical measures such as mean and count of peaks per session. We also extracted **head orientation**, using the USB camera and the tablet camera. After detecting the face region (Zhang et al. 2017), a search for best alignment of the facial landmark is applied using Face Alignment Network (FAN) that extracts these landmarks in an estimated 3D space (Bulat and Tzimiropoulos 2017). This process is performed at every frame, from which we calculate the head orientation (yaw, pitch, and roll). These features were then summarized using statistical measures such as mean and count of peaks per session.

To analyze **body movement and gestures**, we extract body joints from the USB camera which has a wide-angle view suitable for capturing the upper body. We used OpenPose (Cao et al. 2019), which extracts 25 body joints with an estimated 3D space. Similar to the head behavior, the joints were extracted at every frame and were used to compute body orientation (yaw, pitch, and roll) as well as touching behaviors, such as touching the face, the other hand, or the upper body. Then, we summarized these features over each session in terms of mean and count of peaks.

Regarding the **speech transcript** features, we computed two types of features. The first type of features is a set of statistics on word counts per participant: *average*, *maximum*, *minimum*, and *standard deviation*. We extracted these features following the practice of previous works that use the number of words as a proxy of conversational engagement (Ghandeharioun et al. 2019).

### 5.3 Qualitative analysis

Post-study interview transcriptions were analyzed using the thematic analysis method (Braun and Clarke 2006) to extract salient themes. One post-study interview data was lost due to technical failure, and in total, 34 audio transcriptions were analyzed for the post-study interview analysis. Sixteen themes were identified and two annotators coded the transcription data separately to test inter-rater reliability. Cohen’s kappa for inter-rater reliability showed that the two annotators had high agreement on their annotation for the themes and their definitions, *κ* = 0.810. The definitions of each theme and sub-theme are listed in Table 4. There are three themes (***wellness***, ***social robot*** and ***other***), and each theme has several sub-themes.

Participants’ utterances were first coded with theme and sub-theme, and they were further annotated whether they were positive or negative. We often found participants mention both positive and negative aspects of each theme and sub-theme, as our interview questions explicitly requested participants to elaborate on their likes and dislikes of the study experience.

## 6 Results

### 6.1 Overall intervention outcomes

**Fig. 4:**
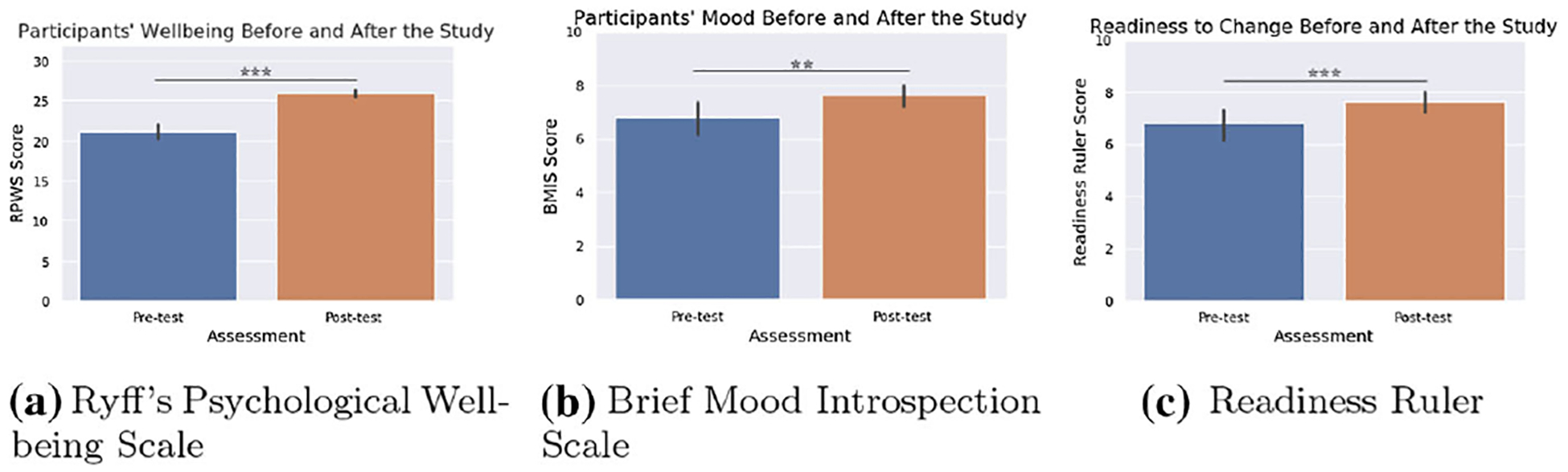
Study participants’ change in psychological well-being, overall mood, and readiness to change behavior before and after interacting with the robotic positive psychology coach

Our previous analyses revealed that completing the seven positive psychology sessions with the robot was associated with students’ increased psychological well-being, mood and readiness to change behavior significantly improved after (Jeong et al. 2020). Students’ psychological well-being (RPWS, max score=42) score was reported as: before *M* = 21.276, SD = 2.540; after *M* = 25.957, SD = 1.529; *t*(34) = −11.843, *p* < 0.001, with a large effect size *d* = 2.233 (Fig. 4a). Students’ mood (BMIS, max score=10) score was reported as: before *M* = 6.800, SD = 1.844; after *M* = 7.629, SD = 1.239; *t*(34) = −3.101, *p* = 0.004, with a medium effect size *d* = 0.528 (Fig. 4b). Students’ readiness to change their behavior for better well-being (Readiness Ruler, max score=10) score was reported as; before *M* = 7.200, SD = 1.132; after *M* = 8.057, SD = 1.371; *t*(34) = −4.170, *p* < 0.001, with a medium effect size *d* = 0.682 (Fig. 4c).

Our robotic coach was able to develop positive working alliance with the students over the seven positive psychology sessions. The overall working alliance score (WAISR, max score=5) was reported as *M* = 3.433, SD = 0.829. The sub-scale *goal* score was *M* = 3.386, SD = 1.033; the *task* score was *M* = 3.100, SD = 0.949; and the *bond* scores was *M* = 3.814, SD = 1.047.

In addition, students in general took longer than seven days to complete the positive psychology sessions with the robot even though they were designed as daily activities. The mean number of days between the first session and the last session were: *M* = 12.229, SD = 8.702, min = 6, max = 45.

### 6.2 Personality traits and intervention outcomes

#### 6.2.1 Students’ personality traits

Students Big Five personality traits and their respective Cronbach alpha values were measured as: extraversion *M* = 2.705, SD = 1.324, *α* = 0.833; agreeableness *M* = 4.148,SD = 1.064, *α* = 0.730; conscientiousness *M* = 3.364, SD = 1.375, *α* = 0.761; neuroticism *M* = 2.841, SD = 1.286, *α* = 0.732; and openness *M* = 2.170, SD = 1.154, *α* = 0.531.

The Shapiro–Wilk test (Shaphiro and Wilk 1965) on each personality trait showed that neuroticism (*W* = 0.968, *p* = 0.389), openness (*W* = 0.947, *p* = 0.094) and conscientiousness (*W* = 0.961, *p* = 0.246) were normally distributed, but extraversion (*W* = 0.933, *p* = 0.034) and agreeableness (*W* = 0.904, *p* = 0.005) were not.

#### 6.2.2 Grouping participants based on personality traits

We investigated the relationship between participants’ personality traits and the efficacy of our positive psychology interventions by dividing participants into (1) high-agreeableness (**A+**) and low-agreeableness (**A−**) groups, (2) high-extroversion (**E+**) and low-extroversion (**E−**) groups, and (3) high-openness (**O+**) and low-openness (**O−**) groups. We also investigated the relationship between personality traits by calculating the Pearson’s correlation coefficients among participants’ Big Five personality traits (Mini-IPIP). Participants’ neuroticism and conscientiousness scores were found to have a statistically significant negative correlation, *r*(33) = −0.418, *p* = 0.013 (Table 5). Based on this finding, we added the fourth analysis pair, (4) low-neuroticism/high-conscientiousness (**N−C+**) and high-neuroticism/lowconscientiousness (**N+C−**) groups. A K-means clustering algorithm was used to generate four pairs of participant groups.

Since not all personality traits were normally distributed, we decided to use the K-means clustering algorithm to group participants based on their Big Five traits. Grouping people based on personality traits have been used in personality psychology in order to identify different types of personality within a population (Sava and Popa 2011; Barbaranelli 2002). Personality-based clustering is also often used in the field of healthcare (Khazaie et al. 2019), e-learning (Tian et al. 2008; Jin et al. 2006), and gaming (Halim et al. 2017) to profile groups of users based on their characteristics and needs for personalized and tailored service and experience.

For the first group pair, 13 participants who have agreeableness scores lower than 4.0 were assigned to the **A−** group and 22 participants whose scores are equal or higher than 4.0 were assigned to the **A+** group. For the second group pair, 22 participants with extroversion scores lower than 3.0 were assigned to the **E−** group and 13 participants whose scores were equal or higher than 3.0 were assigned to the **E+** group. For the third group pair, 17 participants with openness scores lower than 4.0 were assigned to the **O−** group and 18 participants whose scores were equal or higher than 4.0 were assigned to the **O+** group. For the fourth group pair, 21 participants were assigned to the **N+C−** group and 14 participants were assigned to the **N−C+** group. The two centroids were [*N* = 2.381*,C* = 4.095] and [*N* = 3.5*,C* = 2.5], with *N* indicating the neuroticism score and *C* indicating the conscientiousness score respectively.

#### 6.2.3 Ryff’s psychological well-being scale

**Fig. 5:**
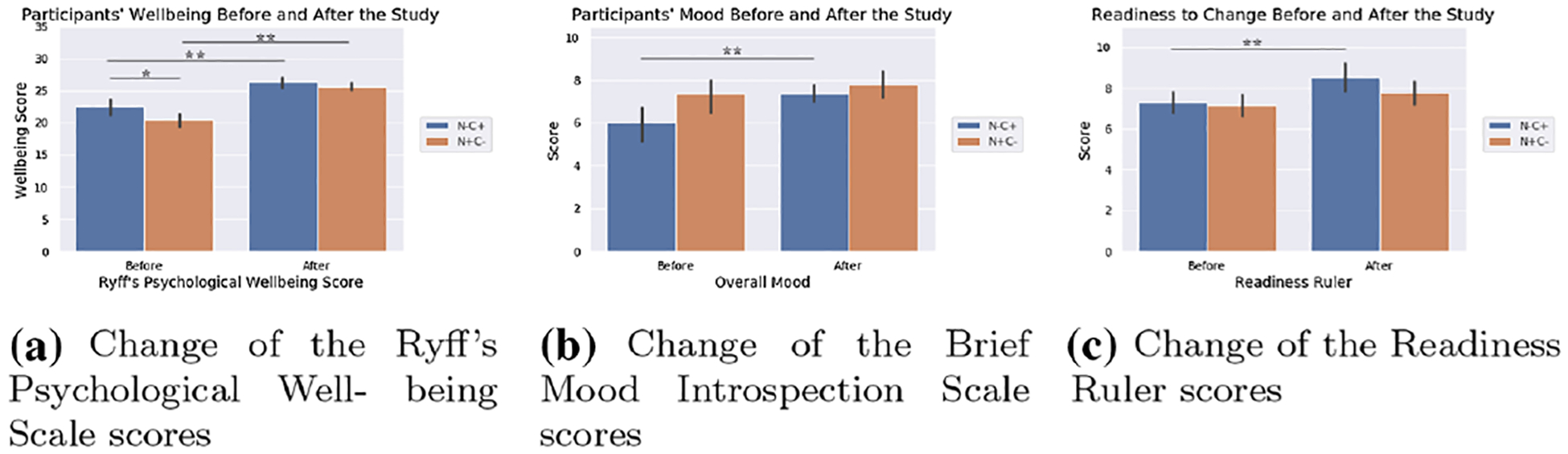
Intervention outcomes based on students’ neuroticism and conscientiousness traits

We found a statistically significant effect of personality traits on the RPWS measures when participants were grouped based on their neuroticism and conscientiousness personality traits. A mixed ANOVA on the pre-to-post comparison of the RPWS showed a significant main effect of participant groups based on personality, *F*(1, 33) = 6.050, *p* = 0.019, *η*2 = 0.155, and a significant effect of time, *F*(1, 33) = 149.208, *p* < 0.001, *η*2 = 0.819 (Fig. 5a). The interaction of time and personality-based groups was found not statistically significant, *F*(1, 33) = 8.148, *p* = 0.084, *η*2 = 0.088. Post hoc Tukey’s test revealed that participants in the **N+C−** group and **N−C+** group both showed statistically significant increase in their psychological well-being after interactingwith the robot: **N+C−** before *M* = 20.444, SD = 2.293; after *M* = 25.683, SD = 1.348; *p* = 0.001, *d* = 2.785; **N−C+** before *M* = 22.524, SD = 2.471; after *M* = 26.369, SD = 1.737; *p* = 0.001, *d* = 1.800. In addition, paired *t* test with Bonferroni correction revealed that the RPWS scores showed statistically significant difference between the two personality groups during the pretest, *t*(26.520) = 2.510, *p* = 0.037, but did not show significant difference in the post test, *t*(23.110) = 1.249, *p* = 0.224. The rest of the group pairs based on other personality traits did not show any statistically significant results on the RPWS measures.

#### 6.2.4 Brief mood introspection scale

There was a statistically significant effect of participants’ neuroticism and conscientiousness traits on the BMIS measures. A mixed ANOVA test on the pre-to-post comparison of the BMIS score revealed a significant effect of time, *F*(1, 33) = 10.107, *p* = 0.003, *η*^2^ = 0.234 (Fig. 5b) but no significant effect on the personality groups, *F*(1, 33) = 3.950, *p* = 0.055, *η*^2^ = 0.107, or the interaction between the time and personality group, *F*(1, 33) = 2.742, *p* = 0.107, *η*^2^ = 0.077. Post hoc Tukey’s test showed the **N−C+** group had significant improvement in their mood after the study: before *M* = 6.000, SD = 1.617; after *M* = 7.357, SD = 0.842; *p* = 0.005, *d* = 1.053. The **N+C−** group did not show any significant improvement: before *M* = 7.333, SD = 1.826; after *M* = 7.810, SD 1.436; *p* = 0.348, *d* = 1.053. The **N+C−** group did not show any significant improvement: before *M* = 7.333, SD = 1.826; after *M* = 7.810, SD = 1.436; *p* = 0.348, *d* = 0.290. The rest of the group pairs based on other personality traits did not show any statistically significant results on the BMIS measures.

#### 6.2.5 Readiness Ruler

Participants’ neuroticism–conscientiousness traits showed significant effect on their Readiness Ruler response. A mixed ANOVA test on the pre-to-post comparison of the Readiness Ruler score showed a significant effect of time, *F*(1, 33) = 17.936, *p* < 0.001, *η*2 = 0.352, but no significant effect of the personality groups, *F*(1, 33) = 1.360, *p* = 0.252, *η*2 = 0.040, or the interaction between time and personality group, *F*(1, 33) = 2.075, *p* = 0.159, *η*2 = 0.159 (Fig. 5c). Post hoc Tukey’s test showed that the **N−C+** group’s readiness to change significantly improved after the study: before *M* = 7.286, SD = 0.994; after *M* = 8.500, SD = 1.345; *p* = 0.007, *d* = 1.027, but the **N+C−** group did not show any significant improvement: before *M* = 7.143, SD = 1.236; after *M* = 7.762, SD = 1.338; *p* = 0.119, *d* = 0.481. The rest of the group pairs based on other personality traits did not show any statistically significant results on the Readiness Ruler measures.

#### 6.2.6 Working Alliance Inventory-Short Revised

**Fig. 6:**
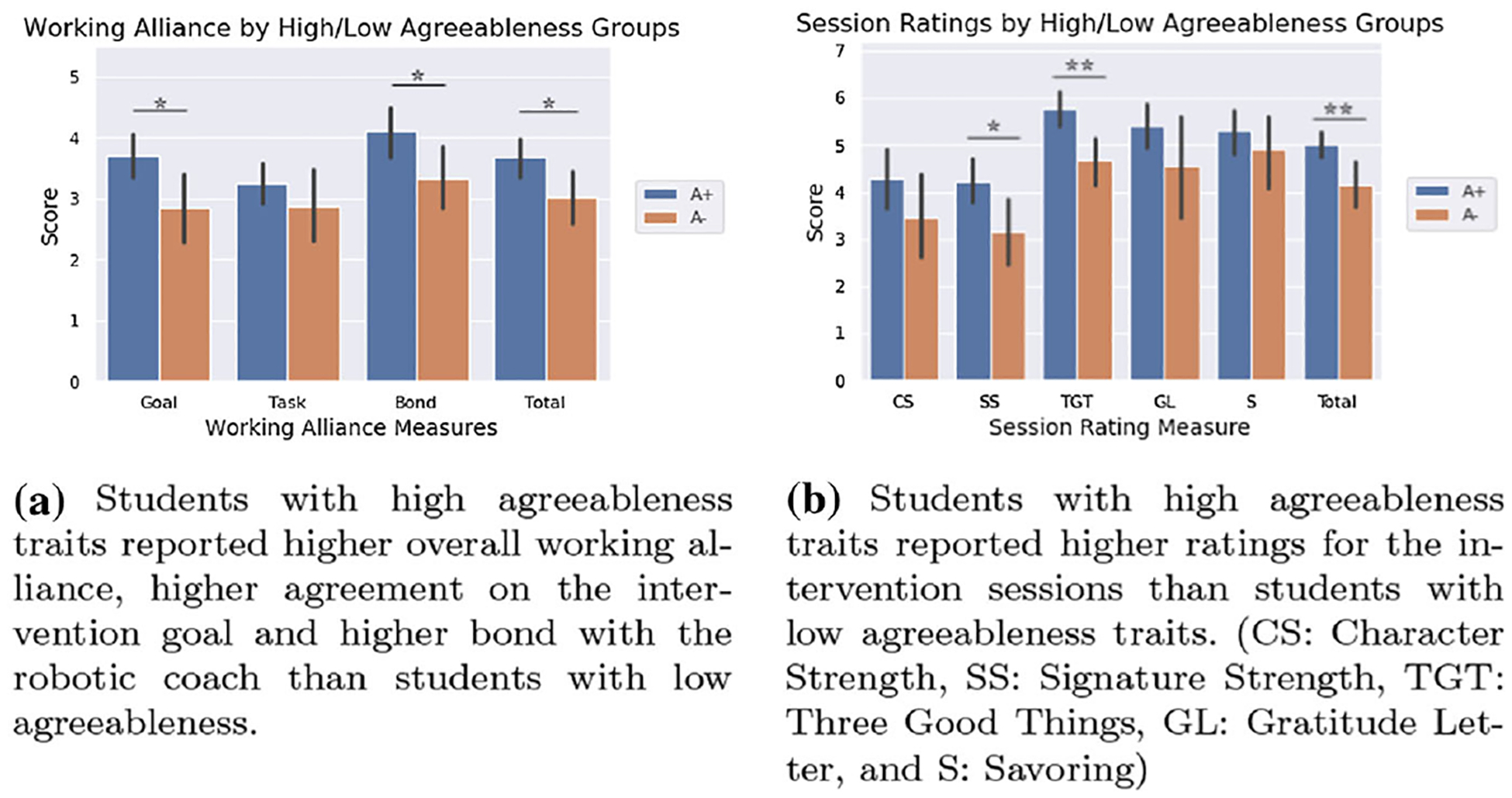
Results based on clustering low-/high-agreeableness personality trait

Participants’ agreeableness showed significant effect on their working alliance with the robotic coach. Student’s *t* tests were conducted to investigate the working alliance of participants in high agreeableness (**A+**) and low-agreeableness (**A−**) group (Fig. 6a). The overall working alliance with the robot was found higher in the high agreeableness group than the low-agreeableness group: **A+** group, *M* = 3.682, SD = 0.746; **A−** group, *M* = 3.013, SD = 0.817; *t*(34) = 2.474, *p* = 0.019, *d* = 0.855.

We found a statistically significant difference in the *goal* and the *bond* subscale scores of the WAI-SR. Regarding the *goal* score, the **A+** group reported *M* = 3.705, SD = 0.889, while the **A−** group reported *M* = 2.846, SD = 1.068, resulting *t*(34) = 2.562, *p* = 0.015, *d* = 0.874. Regarding the *bond* score, the **A+** reported *M* = 4.102, SD = 0.987, while the **A−** group reported *M* = 3.327, SD = 0.997, resulting *t*(33) = 2.237, *p* = 0.032, *d* = 0.781. No statistically significant difference was found in the *task* score between the two agreeableness-based groups: **A+** group, *M* = 3.239, SD = 0.847; **A−** group, *M* = 2.865, SD = 1.097; *t*(34) = 1.128, *p* = 0.267, *d* = 0.382

The rest of the group pairs based on other personality traits did not show any statistically significant results on the WAI-SR scores.

#### 6.2.7 Intervention session ratings

A mixed ANOVA was conducted to compare the ratings of each positive psychology intervention session between high- and low-agreeableness participant groups (Fig. 6b). We found a statistically significant effect of personality group, *F*(1, 33) = 9.765, *p* = 0.004. There was a statistically significant effect of intervention type as well, *F*(4, 132) = 12.366*, p* < 0.001. We did not find any effect on the interaction between the intervention type and the personality-based group, *F*(4, 132) = 0.426, *p* = 0.789.

The post hoc Tukey’s test showed that the high agreeableness group and the low-agreeableness group reported statistically significant difference in ratings for *Using Signature Strength in a New Way* (*SS*) session (**A+** group: *M* = 4.227, SD = 1.165, **A−** group *M* = 3.154, SD = 1.292; *p* = 0.014, *d* = 0.872) and *Three Good Things* (*TGT*) session (**A+** group *M* = 5.773, SD = 0.910, **A−** group *M* = 4.692, SD = 0.910; *p* = 0.001, *d* = 1.188). Ratings for *Character Strength* (*CS*), *Gratitude Letter* (*GL*) and *Savoring* (*S*) sessions did not show any difference between the two participant groups: CS **A+** group *M* = 4.273, SD = 1.543, **A−** group *M* = 3.462, SD = 1.600, *p* = 0.150, *d* = 0.516; GL **A+** group *M* = 5.409, SD = 1.114, **A−** group *M* = 4.538, SD = 1.946, *p* = 0.102, *d* = 0.549; S **A+** group *M* = 5.318, SD = 1.061, **A−** group *M* = 4.923, SD = 1.492, *p* = 0.376, *d* = 0.305.

Overall, the ratings across all sessions between the high and the low-agreeableness groups showed statistically significant difference: **A+** group *M* = 5.000, SD = 0.650, **A−** group *M* = 4.154, SD = 0.898, *t*(19.260) = 2.863, *p* = 0.010, *d* = 1.079.

Across all participants, the pairwise *t* tests with Bonferroni correction revealed that the mean rating for *CS* is significantly lower than themean ratings for*TGT* (*p* = 0.002, *d* = 1.016), *GL* (*p* = 0.023, *d* = 0.697), and *S* (*p* = 0.011, *d* = 0.819). The mean rating for *SS* is also significantly lower than the mean ratings for *TGT* (*p* < 0.001, *d* = 1.278), *GL* (*p* = 0.007, *d* = 0.865), and *S* (*p* < 0.001, *d* = 1.028). No other statistically significant difference was found among other pairs of intervention sessions.

### 6.3 Working alliance and intervention efficacy

Pearson’s correlation coefficients were computed between the four working alliance measures (*WAI-goal, WAI-task, WAI-bond, and WAI-total*) and the three pre-to-post changes in self-report measures (Δ*RPWS*, Δ*BMIS*, and Δ*Readiness*). The results suggest that three out of the twelve correlations were statistically significant. The change in Readiness Ruler*_*Δ*Readiness* between the pre-and-post tests were positively correlated with *WAI-goal*, *WAI-task*, and *WAI-total*: *WAI-goal*, *r* (33) = 0.396, *p* = 0.018; *WAI-task*, *r* (33) = 0.458, *p* = 0.006; *WAI-total*, *r* (33) = 0.413, *p* = 0.014. The rest of the pairs did not show any statistically significant correlations (Table 6).

### 6.4 Interpretation of behavioral cues

As discussed in Sect. 5.2, observations, linguistic (verbal), and nonverbal behaviors were extracted and summarized through statistical features over the sessions for further analyses to reveal correlations to the self-reported intervention outcomes, i.e., RPWS, BMIS, Readiness Ruler, and Working Alliance. The goal is to provide an insight into observable behaviors that could help predict the efficacy of the intervention during the sessions to adapt the robot coach’s strategies in real time. Behaviors that are significantly correlated with the self-report measures are listed in Tables 7, 8, 9, and 10.

#### Change in RPWS:

Analyzing behaviors correlated with the change in the Ryff’s Psychological Well-being Scale (i.e., delta of pre- and post-scores), a few features from facial expression, head orientation, and speech prosody were significantly correlated, which are listed in Table 7. Based on the results of upper lip raise, jaw drop, voiced and unvoiced segment lengths correlations, participants who spoke more had higher improvement in their well-being. Participants who expressed anger, which is inferred from facial activities such as lowered brows, tightened or raised eyelids and tightened lips, had a lower RPWS change. On the other hand, participants who’s gazed down (toward the robot station) had a higher RPWS change. Quality of speech measured by pitch and jitter were also found to be correlated with RPWS. Pitch, also known as the fundamental frequency (F0), which conveys speech prosody, holds a lot of factors such as the state of mind of the speaker (El Ayadi et al. 2011). Jitter (and shimmer), on the other hand, indicates irregularities in speech production, which are correlated with the presence of noise emission and breathiness (Nunes et al. 2010), where high jitter values indicate roughness or breathiness in the voice. The negative correlation of pitch value and the positive correlation of jitter with RPWS could indicate a low and relaxed voice.

#### Change in BMIS:

Mood improvement through BMIS measures is correlated with several behavioral cues, as listed in Table 8. From the labeled observations, it showed a positive correlation in mood improvements with high percentage/proportion of the participant being focused. Similar to the RPWS, expression of negative emotions like anger has a negative correlation with BMIS change. Moreover, less face and upper body touching, which could indicate less stress, were found to positively correlate with mood improvements. Unlike RPWS, looking up was positively correlated with BMIS change, as well as head tilting. Even though this indicates that the participants were not looking at the Jibo station, in conversation context, looking up and head tilting to the right indicates interest and engaged listening (Hadar et al. 1985), which could explain the participant’s mood improvement. For speech, as found with RPWS, relaxed voice and more talking during the session were positively correlated with mood change.

#### Change in Readiness Ruler:

Behaviors correlated with the shifts in readiness to change are listed in Table 9. Similar to the results from RPWS and BMIS, negative emotions such as anger, disgust, and fear (including their action units) were negatively correlated with readiness to change. Moreover, a straight body posture, less self-soothing touches (indicated by touching face, body, and the other hand), as well as relaxed voice were correlated with high readiness to change scores. Participants who gazed at the robot more (looking up and looking left toward the robot) also had higher readiness to change scores. These behaviors indicates that high involvement in the interaction with a relaxed attitude could be predictive to the positive intervention outcome.

#### WAI-SR:

Behaviors correlated with students’ working alliance (WAI-SR) with the robot are listed in Table 10. Participants who had higher average duration of being off-task (including not gazing to the robot station area) had a lower WAI-SR score. Extracted nonverbal behaviors showed that a greater facial expression of attention (measured by straight face orientation), and head orientation (looking up and to the right—away from the robot station) being correlated with a lower WAI-SR score. Furthermore, expression of negative emotion including sadness and action units that typically indicate negative emotions (e.g., eyelid tighten indicating anger or fear) were negatively correlated with rapport. Also, less self-soothing touches, relaxed voice and more speech were positively correlated with a high WAI-SR score. These findings are similar to behavioral analysis of other intervention outcomes described above.

### 6.5 Post-study interview

#### 6.5.1 *Wellness* theme

Table 11 summarizes the thematic coding analysis results. During the post-study interview, all thirty four participants (100%) spoke on the theme of *wellness* because they were explicitly asked questions about the wellness sessions. The *gratitude* exercise was the most frequently discussed type of positive psychology intervention, followed by *savoring*, and *character strengths*. All participants who spoke on the *savoring* and *gratitude* exercises made almost entirely positive comments. For instance, **P06** commented that “*savoring was really cool. I really enjoyed that as well. It’s like not something like I’ve ever done before so it was pretty cool to experience*.” Many participants cited the *gratitude* and *savoring* exercises as being memorable and ones that they would continue after the study (e.g., “*…and like really being aware of yourself as you do activities. So that one [savoring activity] I would like to continue to work on*,” **P11**). This is potentially due to the fact that these exercises were presented in later sessions and were more activity-based.

Participants had more mixed opinions on the *character strengths* intervention. Thirteen participants (38%) made positive comments about it, and eleven participants (32%) spoke negatively about it. Some participants felt as though the *character strengths* exercise was less concrete than the *gratitude* and *savoring* exercises, making it was more difficult to find an actionable goal from the activity. When asked about the wellness activities **P11** stated, “*Uh so I’m not sure if I, if I gained much from me myself going and looking at the strengths and saying what I thought that I had. I feel like the other activities were interesting and they, they made you think more about your day*.”

Participants were also asked explicitly about the theme of *time* and whether they felt that the sessions were an appropriate length for their schedules or if they wanted to spend more or less time on each session. Twenty-one participants (62%) spoke positively about the theme of *time*, generally stating that they felt the length of the sessions felt appropriate and fit with their schedules. **P30** said, “*I feel like they were generally very concise. I think it was, it was a good, good length, yeah. Because any more I feel like I would, yeah, I would mind it a bit if it were a bit too long and even then, like, I know it was very short but some days I would come back to my room so late, like, I wouldn’t have time to do it. Um, so I feel like the length was good, yeah. The length was good*.” However, twenty-two participants (65%) gave negative feedback, often noting that they would have benefited from longer and more in-depth content. P05 stated, “*I almost think, even if they’re a good length now, I think they could have been a little longer, just more, more time, I don’t know. I felt like sometimes I was just like, oh this is cool I wonder what else Jibo will say about it- And she was like ‘That’s all for today’. I was like ‘Oh’. I want to talk more about it*.” **P05**’s statement echoes what many other participants reported. Although they appreciated the short interaction length, they wished more in-depth content was delivered and had hoped for richer interactions with the robot.

#### 6.5.2 *Social robot* theme

The theme of *social robot* was mentioned by thirty three participants (97%). Thirty one of them (91%) spoke positively and ten of them (29%) spoke negatively about the theme. Participants appreciated the robot’s companionship throughout the one-week study. When asked about their ideal robot, many stated that they would want a social robot companion. P07 shared, “*Alexas and Siris they all do specific things- When you ask them to but they don’t actually have conversations. Um. I think it would be cool to have one that can have a conversation like Jibo*.” **P42** noted the impact of having an animate social agent in her room, “*I feel like his [robot’s] presence really did have an effect on how I felt… I felt like just talking to him was useful, having him here was useful… I would like to just be able to talk to it and be able to just communicate ‘cause a lot of times with students you just need someone to talk to sometimes. Like not human being and it’s nice to have a robot that doesn’t judge you (laughs)*.”

Thirty two participants (94%) mentioned the theme of *character* and spoke positively of the robot, describing it as “cute” and “fun” (e.g., “*I think it was very playful. I think that was really fun, actually. Yeah, I really like it*,” **P30**). The robot spoke with a text to speech (TTS) voice and although it was unnatural, most participants were not bothered by it and felt that the robot’s tone and speaking style was appropriate for the wellness sessions.

*Attention* was mentioned by nineteen participants (56%). Seventeen of them (50%) spoke negatively and six of them (18%) spoke positively on the robot’s *attention* behavior. Participants who spoke positively on this theme appreciated how the robot would react to them and felt as though the robot’s movement made them feel less lonely in their rooms. However, those who spoke negatively on this theme noted they wanted the robot to react only when prompted, instead of proactively engaging them in conversations or showing animated idle behavior. **P11** stated, “*I was uncomfortable with how it seems like he’s watching me. That when I’m specifically interacting with him- it’s, it’s nice for him to have those movements. But when I’m just inside my room and he goes and swings towards me. That made me less comfortable*.” Participants often felt disturbed when the robot would suddenly move or look at them. Many were woken up by the robot or felt uncomfortable when the robot would wake before them. Although Jibo has features to sleep or stay still and look away, this was not sufficient for many participants. In the future, extra design considerations should be made for robots being placed in highly personal and intimate settings.

*System* was another theme that received negative feedback from participants. Twenty-eight participants (82%) made negative comments about the robot’s system, and two participants (6%) made positive comments. Lag in Jibo’s response due to the slow wireless network in students’ campus dormitories was the primary reason for the negative feedback. **P17** said, ‘*‘I can’t say there’s too much [dislikes for robot] but he was pretty glitchy. Um, when like loading some of the exercises. Like he’d get stuck and it would, like, lag for like an entire hour before he’d, like, activate, to um… like be… actually like get on with the exercise*.”

#### 6.5.3 *Other* themes

*Security and privacy* was mentioned by eleven participants (32%). Nine participants (24%) spoke negatively on the theme, noting concerns of privacy for themselves and those they share their living spaces with. **P01** stated “*I had a friend over and they were like, paranoid. ‘Jibo is watching us.” (laughs) Yeah. Which is not just all that, like, bad- but like […] it’s a bit disconcerting, but like even though that camera didn’t bother me as much- It bothered my friend more*.” Participants were informed that the robot station would only record video and audio during the wellness sessions, which alleviated concerns for most study participants. However, their roommates and guests often did not share the same confidence in the system’s respect for their privacy.

## 7 Discussion

### 7.1 The robot intervention was associated with students’well-being

In this paper, we present a robotic positive psychology coach designed to improve college students’ psychological well-being. In our prior work (Jeong et al. 2020), we have reported positive association between college students’ psychological well-being, mood and readiness to change for better well-being and our robotic intervention. Results from the WAI-SR and the post-study interviews also suggest that students built positive working alliance and rapport with the robot.

### 7.2 Personality traits are associated with intervention outcomes

We found a statistically significant association between students’ neuroticism/conscientiousness traits and their psychological well-being, mood and readiness to change response (Jeong et al. 2020). While the low-neuroticism and high-conscientiousness (**N−C+**) group showed improvement in all RPWS, BMIS, and Readiness Ruler after the study, the high-neuroticism and low-conscientiousness (**N+C−**) group only showed improved RPWS measure and no significant change in the BMIS and Readiness Ruler measures. **N+C−** group’s RPWS scores were also found to be significantly lower than **N−C+** group’s RPWS scores in the pretest (**H2**). Additional analyses on other personality-based groupings showed that students’ agreeableness, openness, and extraversion did not have a significant impact on the intervention outcomes.

Several studies on personality traits have found high neuroticism negatively correlating to mental health (Heaven et al. 2001; Smith et al. 2017; Lahey 2009). In addition, Smith et al. (2017) report high conscientiousness predicts lower incidence of anxiety disorders and depression, while high neuroticism relates to greater likelihood of these problems. These previous work provides insight to why students with high-neuroticism traits had reduced response to our robot intervention than the students with high-conscientiousness traits. It is also important to note that the **N+C−** group’s pre-study RPWS scores were significantly lower than **N−C+** group’s pre-study RPWS scores. This reflects the work done by Lahey (2009), in which high neuroticism were found to be a predictor for several mental/physical disorders and comorbidity among them. In our future work, we plan to investigate ways to support students with different personalities based on each personality group’s needs, especially for those in the high-neuroticism group.

Students’ agreeableness trait also had a significant association with their ratings for the five positive psychology interventions introduced in the study. Given that students’ agreeableness did not show a significant relationship with intervention outcome, we believe students with high agreeableness trait were more generous and lenient in their evaluation of the intervention content than students with low-agreeableness traits (Bernardin et al. 2000).

### 7.3 Students’working alliance was correlated with motivation for well-being

Our analyses on working alliance (WAI-SR) suggest that the therapeutic alliance between students and robots could impact the intervention outcomes, and students’ personality traits might impact how students bond with the robot. We found a statistically significant association between students’ working alliance and their pre-to-post change in Readiness Ruler response. However, WAI-SR did not show significant correlations with the change in students’ psychological well-being and mood. In addition, we found that students’ agreeableness has a significant impact on the rapport they build with the robotic coach. Our results partially align with the work done by Hirsh et al. (2012), in which patients with high agreeableness traits showed stronger working alliance with their clinicians than patients with low-agreeableness traits, and the intensity of working alliance was associated with better clinical outcomes. Their analysis revealed that working alliance worked as a significant indirect mediator that connects patients’ agreeableness trait to better clinical outcomes. We suspect our results did not show any significant relationship between students’ working alliance and their well-being/mood outcomes due to the short duration of the study. Thus, a follow-up experiment with longer deployment is currently under preparation to study the relationship between human–robot working alliance and the robot intervention outcomes.

### 7.4 Exploratory analyses on human behavioral cues

We conducted a set of exploratory analyses on the participants’ verbal and nonverbal behaviors and their relationship with intervention outcomes. Our results showed that the changes in intervention outcomes were associated with students’ relaxed vocal expression, positive facial expression, and physical engagement behaviors analyzed from head and body gestures. Relaxation is reported to lead to better mental health therapy outcomes including anxiety intervention (Montero-Marin et al. 2018). In some of our sessions, participants were asked to close their eyes and take deep breaths to bring them to a relaxed state of mind for visualization/reflection activities. Participants who exhibited relaxed behaviors in their vocal (relaxed tone of voice), facial (less negative sentiment), motion cues (less self-soothing motions such as touching the face or the body), and engaged behaviors (looking at the robot and engaged listening) responded better to the robot coach sessions and showed higher improvement in their well-being. We further found that users’ affective engagement observed via facial expression and verbal sentiment and behavioral engagement observed via body, head and gaze behavior were correlated with their intervention outcomes. These results echo the work by Short et al. (2018) that digital mental health technologies should incorporate behavioral engagement measurements instead of relying solely on the system usage data (e.g., usage time) for delivering successful interventions. Although our analysis only shows correlations and do not necessarily imply causation, these results suggest certain user behaviors could be informative in predicting the effectiveness of an intervention. Such knowledge will enable a robotic coach to identify whether the interaction is “going well” to result in optimal mental health outcomes, and allow it to adapt its behavior or the intervention content during the interaction.

### 7.5 Considerations for home deployment studies

Qualitative analysis on the post-study interview showed that our robotic coach successfully built rapport and working alliance with our participants through its intervention as well as its other skills. However, the robot’s proactive behavior caused discomfort for some participants. Based on our participants’ feedback, we plan to improve several aspects of our robot system in order to mitigate people’s privacy concerns and discomfort. without compromising the rapport-building experience the robot offers through its animate and life-like behavior.

We suggest users can be given more control over the robot’s “idle” and “proactive” behavior. Our interview results suggest that some participants enjoyed and benefited from robot’s companion-like features, but others felt discomfort from the robot’s attentive behaviors, e.g., following a person’s face, orienting toward a sudden noise, etc. In order to provide more direct control over the robot’s idle behavior, we plan to design a hat-like accessory that physically covers the cameras on the robot and puts the robot to “sleep” when put on. In comparison with a mobile app or a screen-based feature, such physical device could provide an intuitive visual cue on the robot’s status, and would be easy to use for people who are not familiar with screen-based technologies, e.g., older adults. In addition, it would physically cover the cameras on the robot and give additional assurance for users, analogous to webcam covers commonly used on laptop devices.

The robot could be programmed to verbally or visually report the status of data collection upon a user’s request. During the consent process, all of our study participants were notified that the video and audio recordings would only occur during positive psychology session. We also reinforced this information verbally during the initial setup process. However, many students still reported feeling unsure when the robot was recording or not during the post-study interview sessions. These suggest that long text-based information on the data collection might not be the best method to inform users on how their data are being collected, used and stored. Thus, we propose implementing a feature in the robot to report what sensors are being used and how the collected data are processed, recorded and stored in layman’s terms upon users’ request, e.g., “Right now, I’m using my cameras to find your face and my microphones to hear what you are saying.”

The number of recording devices deployed on the robot system could be reduced and the amount of recorded raw data can be minimized in order to respect people’s privacy. In this study, we decided to install static cameras to capture students’ interactions with the robot in order to study the fine-grained behavioral cues during the human–robot interaction. However, when participants were not actively engaging in the positive psychology sessions, video/audio data were processed in real time for the robot to react and respond to participants and were discarded afterward. We plan to use the recorded interaction data to develop computational models that can detect behavioral cues that signal users’ affect and rapport with the robot. Once such model is developed, we plan to deploy the robot that use the data from the sensors to detect various behavioral cues but do not store any raw footage.

Participants’ feedback on the positive psychology sessions and the robot suggests the need to further personalize the robot’s behavior and interventions. We recommend future researchers to design robot-mediated mental health interventions in ways that empower users to tailor how they interact with the robot based on their lifestyle, needs, and preferences with flexibility and adaptability. For instance, some students might want to engage in short 5–10-min sessions with the robot to quickly debrief at the end of everyday, or others might prefer longer interactions at a time with lower frequency. Instead of providing a one-size-fits-all interactions, enabling users to continuously adapt and adjust how they want to be supported through these interactive AI technologies would be important for future research.

### 7.6 Limitations

Our study was exploratory in its nature and did not include a control group who did not receive any robot intervention. Thus, we cannot argue students’ improved self-report outcomes were caused solely by the interactions with our robotic coach. It is possible that the robot’s presence alone could have made a difference, or administrating the well-being scales could have led to changes in students’ well-being status (e.g., Hawthorne effect Jones 1992). However, we would like to note that it is very unusual to observe significant improvement in college students’ well-being during an academic term. The SNAPSHOT study (Sano 2016) has shown that students’ well-being generally declines over the course of a semester. Our participants started the study during the first half of a Fall semester and completed it in the latter half of the semester. Thus, our participants’ improvement in psychological well-being goes against the typical trajectory (Sano 2016), and this suggests that the robot coach’s positive psychology interactions likely played a positive role in the improvement in students’ well-being.

We also did not have a group of participants who were given a robot with only the positive psychology activities or only the built-in skills. We plan to run a follow-up study to compare the effect of (1) the robot with standard skills only, (2) the robot with positive psychology activities only, and (3) the robot with both standard skills and positive psychology activities to further investigate the role of non-therapeutic interactions on the mental health intervention outcomes.

Lastly, our data suffer from common challenges of uncontrolled and noisy data collected in the real world. For example, sometimes the face of the participant was not completely visible, or the face was captured from a very low angle, causing important image deformations. At times, the room had very low illumination and the acquired videos were dark. These challenges can affect the performance of the automatic models used to infer the behavior features. Moreover, even in optimal conditions the current automatic models to infer behavior features are not always accurate. Thus, the verbal and nonverbal behavior correlation results we report in Sect. 6.4 should be considered in limited context and should not generalized to different settings. However, it is interesting to notice that we observed some repetitive patterns of behavior correlation across RPWS, BMIS, Readiness Ruler, and WAI-SF outcomes. Relaxed voice, positive facial sentiment, and engaged behaviors are the most observed ones, which were in line with research on behaviors related to successful mental therapies.

### 7.7 Summary

Our deployment study with college students demonstrated that unlike other existing health technologies, a social robot can create unique opportunities to build rapport with its users through pro-social behaviors. Our novel intervention that offers both positive psychology based interactions and companionship allowed the alliance built between students and the robot to further improve the effectiveness of its positive psychology sessions. Although not explored in this paper, we believe the working alliance between the agent and students could further support students’ adherence to the mental health interventions in long-term therapy context. Huddy et al. (2012) showed that clients’ working alliance and satisfaction have significant effect on how long they stay in therapy and improve toward their target mental health goals. We further identified several factors that can impact the intervention outcomes. Based on our results, students’ personality traits as well as their behavioral cues during the interactions can provide insightful information on the effectiveness of the intervention. Current digital mental health interventions still struggle to keep users engaged in the intervention over an extended period of time. Our findings offer valuable insights to personalize and tailor health technologies to improve user satisfaction and retention as well as intervention efficacy.

## 8 Conclusion

Current interactive technologies for well-being, including chatbots, virtual agents, and conversational agents, offer limited opportunities to build rapport with users. The results from these well-being intervention technologies show mixed results, which hinder the ability to generalize their protocol. In this paper, we present a robotic positive psychology coach that can be deployed to on-campus dormitories and interact with college students to improve their well-being. In our deployment study, 35 college students lived with the robotic coach while completing seven positive psychology sessions and had opportunities to build rapport with the robot through the intervention session as well as its other useful skills and proactive behavior throughout the day.

We found that college students’ interactions with our robot were associated with their improved psychological well-being, mood, and readiness to change behavior for better well-being. We also present important and novel observations on how students’ personality traits, working alliance with the robot, and behaviors during the interaction are associated with the effectiveness of the robot intervention. Post-study interview data suggest that the robot’s companionship and proactive behavior were positively received by the students. The interview also revealed students’ concerns for privacy and opportunities to improve the transparency of the robot’s data collection and usage in future studies.

Our findings suggest that a social robot could potentially enhance the mental health outcomes by personalizing its long-term mental health interventions, and such personalization can be made based on people’s traits and behavioral cues observed during interactions. In addition, we show it is important to design an interactive agent as a helpful and supportive companion that can build long-term rapport and therapeutic alliance in order to improve the efficacy of its health interventions.

## Figures and Tables

**Table 1:** Descriptions of the seven positive psychology sessions

#	Session name	Description
1	Positive psychology (Seligman and Csikszentmihalyi 2014)	Introduce positive psychology and learn how to interact with the robot
2	Character strengths (Niemiec 2013)	Introduce what character strengths are and identify one’s own signature strengths
3	Signature strength in a new way (Proyer et al. 2015)	Pick one signature strength and think of a new way to use it to improve well-being
4	Three good things (Proyer et al. 2014)	Define what gratitude is and write down three things that went well and why they were positive
5	Gratitude letter (Seligman et al. 2005)	Write a letter of gratitude to someone who has not been properly thanked
6	Savoring (Smith and Hanni 2019)	Choose a small moment to fully feel and appreciate experiences that one normally hurries through
7	Wrap-up	Review the previous sessions, evaluate each intervention, and encourage continuation of practicing the interventions

**Table 2:** List of during pre-, mid-, and post-study measures

Time	Measures
Pre-study	Mini-IPIP
	Ryff’s Psychological Well-being (RPWS)
	Brief Mood Introspection Scale (BMIS)
	Readiness Ruler
Mid-study	Video/audio recording of each wellness session
Post-study	Ryff’s Psychological Well-being (RPWS)
	Brief Mood Introspection Scale (BMIS)
	Readiness Ruler
	Working Alliance Inventory-Short Revised (WAI-SR)
	Semi-structured Interview

**Table 3:** List of post-interview questions

#	Question
1	What was it like having Jibo in your home/room?
2	Describe to me your interactions with Jibo
3	What was three things you liked about your interactions with Jibo?
4	What was three things you disliked about your interactions with Jibo?
5	What other things did you do with Jibo other than the wellness skill? What did you like/dislike about them?
6	What did you think of the wellness activity content?
7	What did you think about the length of each session and the amount of content presented? Did you find them interesting or boring? Why?
8	What did you think of the robot’s personality and talking style? Too formal or casual? Why?
9	Would you continue practicing the wellness activities you learned from Jibo even after the robot is no longer with you?
10	Let’s imagine a world where everyone has a personal robot like we have smartphones now. If there’s no restriction in technology, resources, etc., what would you like the robot do for you? What would be your “dream” robot?

**Table 4:** Definitions of each theme in post-interview analysis

Theme	Definition
Wellness	This is the first level of classification. If the comment is about any aspect of the wellness intervention’s content, you label it as “wellness.” Aspects of the wellness content may include the general structure of each daily session or the week-long program
Character Strengths	This is a subcategory of Wellness. It refers to comments made about the character strength activities or topic taught in the interventions. (Picking your character strengths, and using one in a new way)
Savoring	This is a subcategory of Wellness. It refers to comments made about the savoring activities or topic taught in the interventions. (Picking an activity to savor, and reflecting on the activity)
Gratitude	This is a subcategory of Wellness. It refers to comments made about the gratitude activities or topic taught in the interventions. (3 good things and the gratitude letter)
Time	This code is a subcategory of wellness and refers to comments on the duration of the wellness sessions. It also may refer to how the sessions integrated with the participant’s overall schedule
Social robot	This code refers to comments about robots exhibiting social behaviors or limitations in the ability to express these behaviors. Such behaviors may include holding conversation and listening, acting as a companion, and the robot’s ability to comprehend or adapt to what the user is saying or doing (personalization and natural language processing). Generally the type of conversation extends beyond basic command and response. Instead they include turn-taking conversations that humans might hold
Character	This code refers to comments around the robot’s character. Such traits might include the robot’s personality as well as its physical form/embodiment, style of speaking, and tone of voice
Attention	This code refers to comments about the robot’s responsiveness to the participant. Indicators of attention might include motion, sleep and wake states, and attentiveness to the participant. This does not include issues caused by lagging
Learning	This code largely refers to comments about learning or thinking abouttopics in a new way. It may cover the participant learning on their own, or the robot helping to guide or teach them new things
Utility	This code refers to participants’ comments about convenience and utility-based features or concepts. Utility-based features are considered those in which the participant is asking the robot to do something for them, not with them. Utility interactions are transactional in nature. Examples include information-based queries, weather, music, etc.
Entertainment	This code refers to comments about entertainment. Such discussion may revolve around specific features of the robot, or more generally the robot as a platform for entertainment. Entertainment features are defined as those that are used for fun and they may be novel to the robot. These features are more relational and tend to provide an experience rather than a utile service
System	This code refers to comments about the interaction of the robot with either the tablet, network, wifi, etc. Concerns about connectivity and lagging are examples of “system”
Security and Privacy	This code refers to comments about security, privacy, data collection, information handling, data transparency, etc.
Devices	This code refers to mentions of other robot or voice agent technologies other than the robot being used in the study
Social	This code refers to comments about the participant’s social environment. This includes their living-space, their friends/others in their lives and the positive or negative changes in the environment due to the robot or other reasons
Mood	This code largely refers to comments about the participant’s emotional state throughout the time with the robot. How the participant was feeling

**Table 5:** Correlations among participants’ personality traits based on Mini-IPIP assessment

Personality 1	Personality 2	*r*-coefficient	*p* value
Extroversion	Openness	*r* (33) = −0.020	0.907
	Agreeableness	*r* (33) = 0.235	0.175
	Conscientiousness	*r* (33) = −0.020	0.908
	Neuroticism	*r* (33) = 0.065	0.712
Openness	Agreeableness	*r* (33) = 0.310	0.070
	Conscientiousness	*r* (33) = 0.111	0.526
	Neuroticism	*r* (33) = −0.188	0.280
Agreeableness	Conscientiousness	*r* (33) = 0.080	0.650
	Neuroticism	*r* (33) = 0.121	0.487
Conscientiousness	Neuroticism	*r* (33) = −0.418	0.013*

The bold with one asterisk indicates that the *p* value is < 0.05

**Table 6:** Correlations between participants’ working alliance and the pre-to-post change in self-reported intervention outcomes

WAI measure	Pre-to-post outcome change	*r*-coefficient	*p* value
*WAI-goal*	Δ*RPWS*	*r* (33) = −0.214	0.218
	Δ*BMIS*	*r* (33) = −0.084	0.630
	Δ*Readiness*	*r* (33) = 0.396	0.018*
*WAI-task*	Δ*RPWS*	*r* (33) = −0.130	0.456
	Δ*BMIS*	*r* (33) = 0.100	0.568
	Δ*Readiness*	*r* (33) = 0.458	0.006**
*WAI-bond*	Δ*RPWS*	*r* (33) = −0.270	0.116
	Δ*BMIS*	*r* (33) = −0.224	0.196
	Δ*Readiness*	*r* (33) = 0.175	0.315
*WAI-total*	Δ*RPWS*	*r* (33) = −0.252	0.144
	Δ*BMIS*	*r* (33) = −0.091	0.602
	Δ*Readiness*	*r* (33) = 0.413	0.014*

The bold with one asterisk indicates that the *p* value is < 0.05 and the bold with two asterisks indicates that the *p* value is < 0.01

**Table 7:** Behaviors correlated with the change in the Ryff’s psychological well-being scale (RPWS) (only statistically significant results are listed)

	Behavior	*r*(33)	*p* value	Stats
Labeled behavior	–			
Facial expression	Avg. anger	− 0.231	0.004	Spearman
	Avg. Jaw drop	− 0.184	0.024	Spearman
	Avg. upper lip raise	0.199	0.015	Spearman
Body movement	–			
Head orientation	Avg. head pitch	− 0.297	0.001	Spearman
Speech prosody	Avg. unvoiced segment	− 0.119	0.000	Spearman
	Avg. voiced segment	0.2	0.000	Spearman
	Avg. pitch	− 0.084	0.003	Spearman
	Avg. jitter	0.071	0.013	Spearman

**Table 8:** Behaviors correlated with mood improvement through BMIS measures (only statistically significant results are listed)

	Behavior	*r*(33)	*p* value	Stats
Labeled behavior	Proportion of being focused	0.369	0.037	Spearman
Facial expression	Avg. Anger	− 0.25	0.002	Spearman
	Avg. brow raise	0.269	0.001	Spearman
	Avg. eye widen	− 0.164	0.045	Spearman
Body movement	Avg. right hand to face distance (higher means less touching of face)	0.199	0.03	Pearson
	Avg. right hand to upper body distance (higher means less touching of upper body)	0.255	0.005	Spearman
Head orientation	Avg. head pitch	0.175	0.032	Pearson
	Avg. head roll	0.3	0.000	Spearman
Speech prosody	Avg. pitch	− 0.183	0.000	Spearman
	Avg. jitter	0.075	0.009	Spearman
	Avg. loudness	− 0.13	0.000	Spearman
	Avg. unvoiced segment	− 0.133	0.000	Spearman
	Avg. voiced segment	0.105	0.000	Spearman

**Table 9:** Behaviors correlated with readiness to change (only statistically significant results are listed)

	Behavior	*r*(33)	*p* value	Stats
Labeled behavior	–			
Facial expression	Avg. anger	− 0.277	0.001	Spearman
	Avg. disgust	− 0.302	0.000	Spearman
	Avg. fear	− 0. 268	0.001	Spearman
	Avg. brow furrow	− 0.179	0.029	Spearman
	Avg. brow raise	0.226	0.006	Spearman
	Avg. dimpler	− 0.187	0.022	Spearman
	Avg. lip press	− 0. 17	0.038	Spearman
	Avg. lip stretch	− 0.175	0.032	Spearman
	Avg. mouth open	− 0. 215	0.008	Spearman
Body movement	Avg. body pitch	0.266	0.003	Spearman
	Avg. body roll	0.204	0.025	Spearman
	Avg. body yaw	0.204	0.025	Spearman
	Avg. left hand to face distance	0.293	0.001	Pearson
	Avg. right hand to face distance	0.31	0.001	Pearson
	Avg. left hand to upper body distance	0.257	0.005	Pearson
	Avg. right hand to upper body distance	0.298	0.001	Spearman
	Avg.distance between hands	− 0.349	0.000	Spearman
Head orientation	Avg. head pitch	0.267	0.001	Pearson
	Avg. head yaw	− 0.197	0.016	Pearson
Speech prosody	Avg. shimmer	0.059	0.038	Spearman

**Table 10:** Behaviors correlated with WAI score (only statistically significant results are listed)

	Behavior	*r*(33)	*p* value	Stats
Labeled behavior	Avg. of being off-task	− 0.353	0.048	Spearman
Facial expression	Avg. sadness	− 0.198	0.015	Spearman
	Avg. attention	− 0.322	0.000	Spearman
	Avg. brow raise	− 0.199	0.015	Spearman
	Avg. lid tighten	− 0. 165	0.043	Spearman
	Avg. lip pucker	− 0.185	0.023	Spearman
	Avg. upper lip raise	− 0. 161	0.049	Spearman
Body movement	Avg. right hand to face distance	0.184	0.044	Pearson
	Avg. right hand to upper body distance	0.244	0.007	Spearman
Head orientation	Avg. head pitch	0.288	0.000	Pearson
	Avg. head yaw	− 0. 169	0.039	Pearson
Speech prosody	Avg. loudness	− 0.178	0.000	Spearman
	Avg. unvoiced segment	0.078	0.006	Spearman
	Avg. voiced segment	− 0.085	0.003	Spearman

**Table 11:** Number of participants that mentioned each theme and sub-theme in the post-study interview

Theme	Subtheme	Total	Positive	Negative
Wellness		34 (100%)	33 (97%)	28 (82%)
	Character strengths	20 (59%)	13 (38%)	11 (32%)
	Savoring	20 (59%)	18 (53%)	2 (6%)
	Gratitude	26 (76%)	25 (74%)	2 (6%)
	Time	34 (100%)	21 (62%)	22 (65%)
Social robot		33 (97%)	31 (91%)	10 (29%)
	Character	32 (94%)	32 (94%)	26% (9)
	Attention	19 (56%)	18% (6)	50% (17)
	Learning	20 (59%)	16 (47%)	11 (32%)
	Utility	27 (79%)	25 (74%)	7 (21%)
	Entertainment	30 (88%)	27 (79%)	5 (15%)
	System	28 (82%)	2 (6%)	28 (82%)
Security and privacy		11 (32%)	3 (9%)	8 (24%)
Devices		10 (29%)	9 (26%)	3 (9%)
Social		19 (56%)	16 (47%)	3 (9%)
Mood		7 (21%)	5 (15%)	5 (15%)

## Footnotes

1
https://woebothealth.com/.

2
https://www.headspace.com/.

3
https://www.calm.com/.

4
https://www.jibo.com/.

5
https://firebase.google.com/docs/cloud-messaging.

6
https://socket.io/.

7
https://firebase.google.com/docs/database.

8
https://aws.amazon.com/s3/.
